# Uniform-thickness electrospun nanofiber mat production system based on real-time thickness measurement

**DOI:** 10.1038/s41598-020-77985-0

**Published:** 2020-11-30

**Authors:** Hyun Il Ryu, Min Seok Koo, Seokjun Kim, Songkil Kim, Young-Ah Park, Sang Min Park

**Affiliations:** 1 School of Mechanical Engineering, Pusan National University, Busan, 46241 South Korea; 2 Division of Cardiology, Inje University, Busan Paik Hospital, Busan, 47392 South Korea

**Keywords:** Techniques and instrumentation, Biomaterials, Mechanical engineering, Nanoscience and technology

## Abstract

Electrospinning is a simple versatile process used to produce nanofibers and collect them as a nanofiber mat. However, due to bending instability, electrospinning often produces a nanofiber mat with non-uniform mat thickness. In this study, we developed a uniform-thickness electrospun nanofiber mat (UTEN) production system with a movable collector based on real-time thickness measurement and thickness feedback control. This system is compatible with a collector with void regions such as a mesh-type collector, two-parallel-metal-plate collector, and ring-type collector, which facilitates the measurement of light transmittance across the produced nanofiber mat during electrospinning. A real-time measurement system was developed to measure light transmittance and convert it to the thickness of the nanofiber mat in real time using the Beer–Lambert law. Thickness feedback control was achieved by repeating the following sequences: (1) finding an optimal position of the movable collector based on the measured thickness of the nanofiber mat, (2) shifting the collector to an optimal position, and (3) performing electrospinning for a given time step. We found that the suggested thickness feedback control algorithm could significantly decrease the non-uniformity of the nanofiber mat by reducing the standard deviation by more than 8 and 3 times for the numerical simulation and experiments, respectively, when compared with the conventional electrospinning. As a pioneering research, this study will contribute to the development of an electrospinning system to produce robust and reliable nanofiber mats in many research and industrial fields such as biomedicine, environment, and energy.

## Introduction

Electrospinning is a simple versatile process used to produce nanofibers and interwoven fiber mats^1–4^. Because the nanoscale characteristic length of electrospun nanofibers affords excellent properties such as high porosity, large active surface area, large surface-to-volume ratio, high mechanical performance^5^, and low density^6^, electrospun nanofiber mats have been widely applied in various research fields, including tissue engineering^7–9^, drug release^10^, filtration^11–13^, water management^6^, heavy metal detection^14^, energy storage^15^, energy harversting^16^, reinforcements^17^, actuator^18^, and thermal insulation^19^. An electrospinning configuration primarily consists of a high-voltage supplier, metal needle connected to a syringe and pump, and metal collector. With the ejection of a solution jet through a metal needle, applying a high voltage between the metal needle and the metal collector results in the reduction of the diameter of the solution jet, thereby forming fibers with a nanoscale diameter. Such an application of high voltage induces electrical repulsion and the resulting bending instability^2^, which scatters electrospun nanofibers on the metal collector. This instability of the electrospinning process results in the non-uniform deposition of electrospun nanofibers on the metal collector. Furthermore, because electrospun nanofibers spread through a single metal needle, their deposition is generally concentrated near the closer region of the metal collector from the metal needle compared to the far region. Moreover, multiple needle electrospinning^20^ and needleless electrospinning^21^, which were designed for mass-production of nanofiber mats, also involved the production of non-uniform thickness nanofiber mat thicknesses to a certain extent. This non-uniformity would deteriorate the functionality of the nanofiber mat as a filter or scaffold, as it could affect the filtering efficiency or mechanical properties. Thus, it is desirable to develop an electrospinning process or system that can produce uniform-thickness nanofiber mats for academic and industrial purposes.

Electrospun nanofiber mats have been produced with various electrospinning configurations using different types of collectors^22–24^. The most common type of collector is a flat metal collector or rotating drum^25,26^ that produces a nanofiber mat conformally contacted to the collector. For filter and biomedical applications, separating the nanofiber mat from the flat metal collector or rotating drum is necessary for mass transport through the nanofiber mat^11,27^. Recently, the direct utilization of the nanofiber mat as a filter without separating it from the collector has been achieved by introducing a metal collector with void regions, such as a metal mesh-type collector^28–30^ and two-parallel-metal collector^31,32^, in the electrospinning configuration. Because mass transport is possible through the void region of these collectors, an electrospun nanofiber mat suspended on the void region can be directly utilized as an air or liquid filter. Moreover, electrospun nanofibers were deposited slowly on the void region, and thus, the thickness of the nanofiber mat could be more precisely controlled, thereby achieving a transparent filter with superior performance^28^ or an ultrathin nanofiber membrane mimicking tissue membranes^33,34^. However, collectors with void regions also resulted in the production of non-uniform-thickness nanofiber mats, similar to the conventional electrospinning configuration.

In this study, we propose a uniform-thickness electrospun nanofiber mat (UTEN) production system to fabricate a uniform-thickness nanofiber mat on a two-parallel-metal-plate collector with a void region as a representative example. A two-parallel-metal-plate collector was used because it could be considered as a simple 1D model to deposit a nanofiber mat. To fabricate a uniform-thickness nanofiber mat, we measured the thickness of the nanofiber mat in real time during electrospinning and applied a feedback control algorithm to modulate the deposition of electrospun nanofibers, thereby reducing the non-uniformity in the thickness of the nanofiber mat. However, most previous thickness measurement techniques employing a micrometer screw gauge or cross-sectional image could not measure the thickness of the nanofiber mat in real time during electrospinning. Thus, we developed a real-time thickness measurement system that evaluates the light transmittance of the nanofiber mat based on the light intensity of each pixel in a CCD camera during electrospinning and estimates corresponding thicknesses using the Beer–Lambert law. Based on the feedback control of the estimated thicknesses, the collector was automatically located at an optimal position to reduce the non-uniformity of the nanofiber mat, thereby producing a uniform-thickness nanofiber mat featuring the desired thickness. Finally, we numerically and experimentally confirmed the production of a uniform-thickness nanofiber mat using the proposed UTEN production system.

## Experimental section

### Uniform-thickness electrospun nanofiber mat (UTEN) production system

**Figure 1:**
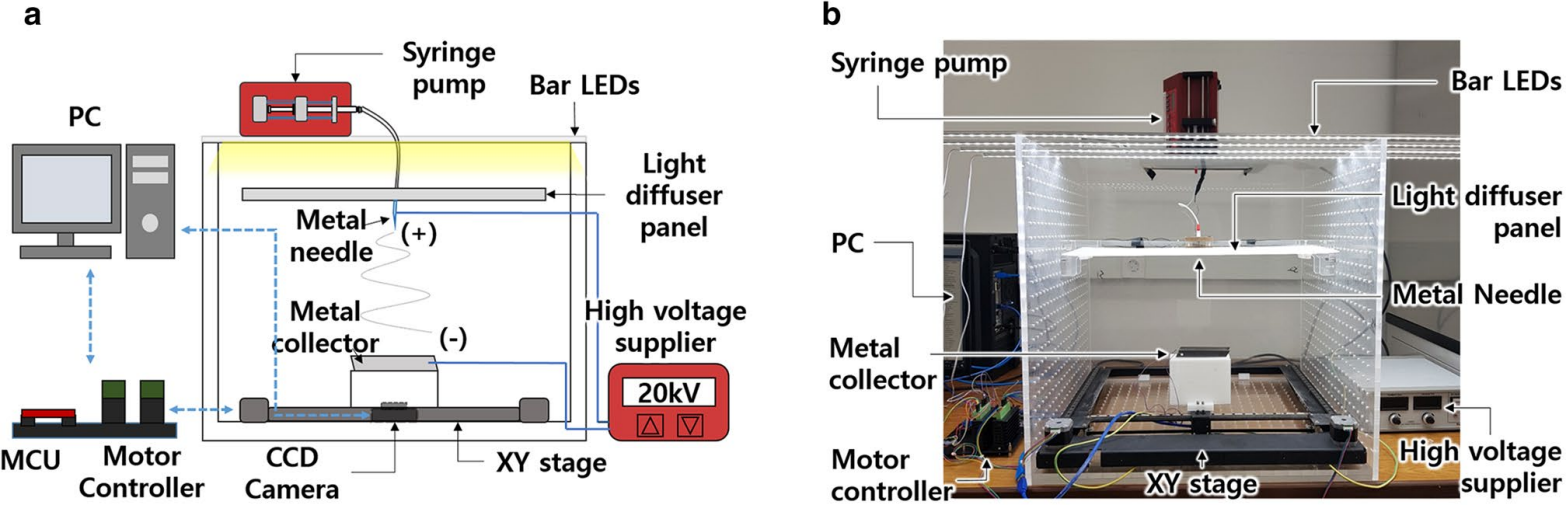
(**a**) Schematic and (**b**) photograph of the uniform-thickness electrospun nanofiber mat (UTEN) production system.

The schematic and photographs of the custom-built UTEN production system are shown in Fig. 1a,b, respectively. The UTEN production system consists of a nanofiber generation system, movable collector, real-time thickness measurement system, and thickness feedback control system. The nanofiber generation system comprises a conventional electrospinning configuration with a high-voltage supplier (HV30, NanoNC, South Korea), syringe pump (Syringe Pump-NE300, New Era, USA), and 5-ml plastic syringe connected to a 23-gauge metal needle with a poly(methyl methacrylate) (PMMA) box. The syringe pump was placed on top of the PMMA box, and the metal needle was fixed in the middle of the PMMA box. The movable collector, installed at the bottom of the PMMA box, consists of a two-parallel-metal-plate collector, a 3D-printed collector holder, and an XY linear stage. The real-time thickness measurement system includes four bar LEDs (PCBNO17003, Daekyung LED Co., South Korea), a PMMA light diffuser panel, and a CCD camera (oCam-1CGN-U-T, WITHROBOT, South Korea). To uniformly illuminate the nanofiber mat, the LEDs were placed on top of the PMMA box with an equal spacing of 5 cm, and the light diffuser panel was located 15 cm below the LEDs (Fig. S1). The light emitted from the LEDs diffuses through the light diffuser panel, uniformly illuminating the nanofiber mat. The light transmitted through the nanofiber mat was measured using the CCD camera placed at the bottom. Based on the measured thickness, the thickness feedback control system actuated the movable collector with a microcontroller unit and PC with custom-built software.

### Electrospinning to fabricate a nanofiber mat

Electrospinning was performed by employing previously reported conditions^32^. A 7.5% polycaprolactone (PCL, Mn = 80,000 g mol^−1^) solution was prepared by dissolving PCL pellets (Sigma-Aldrich, USA) in a mixture of methanol (Samchen Chemical, South Korea) and chloroform (Sigma-Aldrich, USA) for 24 h. The PCL solution was loaded in a 5-ml plastic syringe connected to a 23-gauge metal needle. Using a syringe pump, the PCL solution was ejected through the metal needle at a constant flow rate of 1.5 mL h^−1^. By applying a high voltage of 20 kV between the metal needle and two-parallel-metal-plate collector, electrospun nanofibers were deposited on the collector, forming a nanofiber mat. Electrospinning was performed in a PMMA box in which the humidity was controlled between 40–50% at 25 °C.

### Characterization of a nanofiber mat

The nanostructure of the nanofiber mats was observed at a high magnification of × 800 using scanning electron microscopy (SEM; Supra 45, Carl Zeiss, Germany). Before obtaining an SEM image, the nanofiber mat was sputter-coated with platinum. The thickness of the nanofiber mat was measured using a previously reported method^24^. The nanofiber mat was immersed in a mixture of PDMS monomer and curing agent (Dow Corning Inc., USA) at a weight ratio of 10:1 and baked in a dry oven at a moderate temperature of 50 °C for 24 h. The PDMS-embedded nanofiber mat was cross-sectioned (Fig. S2), and the thickness of the nanofiber mat was measured based on the cross-sectional image captured by a microscope (Zeiss Axio Lab.A1, Zeiss, Germany).

### Conversion from light transmittance to thickness

The light transmittance of the nanofiber mat was converted to its thickness based on the Beer–Lambert law, i.e., $T={e}^{-at}$, where $T$ is the light transmittance, $t$ is the thickness of the nanofiber mat, and $a$ is the attenuation (or extinction) coefficient. To calculate the attenuation coefficient, $a$, five nanofiber mats with different electrospinning times of 15, 30, 45, 60, and 75 min with a time interval of 15 min were fabricated. We measured the light transmittance and thickness of each nanofiber mat at eight equally spaced points. As the conventional electrospinning process frequently produced a non-uniform nanofiber mat, we obtained eight different thicknesses and light transmittances for each nanofiber mat from the eight equally spaced points. The attenuation coefficient, $a$, was calculated from the measured light transmittance and thickness.

### UTEN production simulation software

A simulator software for the UTEN production system was designed based on the deposition behavior of the nanofiber mat on the two-parallel-metal-plate collector. The deposition behavior of the nanofiber mat was assumed to be a Gaussian distribution with an altered center and standard deviation, which was experimentally confirmed. Using the experimentally acquired value of the center and standard deviation and a random number generator, the software simulated the deposition of a nanofiber mat produced by the UTEN production system. Thus, a bottom-view image of the nanofiber mat was generated similar to the image obtained by the CCD camera.

## Results and discussion

### Nanofiber mat production

**Figure 2:**
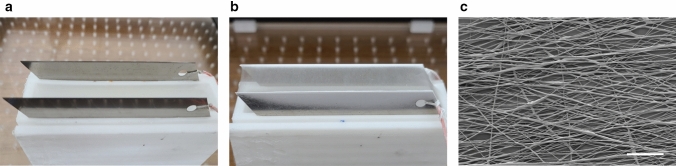
Two-parallel-metal-plate collector (**a**) before and (**b**) after electrospinning. (**c**) SEM image of nanofiber mat. The scale bar indicates 20 µm.

In the electrospinning configuration, the application of a high voltage between the metal needle and the collector induces Taylor cone at the tip of the metal needle, and a polymer jet is ejected at the end of the Taylor cone. Owing to the applied high voltage, the polymer jet carries high surface charges, which cause electrostatic repulsion and subsequent bending instability. The electrostatic repulsion and solvent evaporation of the polymer jet decrease the polymer jet diameter to the nanoscale, thereby forming nanofibers. The resultant nanofibers from the polymer jet are randomly deposited on the collector owing to the bending instability. In this study, a two-parallel-metal-plate collector was utilized to accumulate electrospun nanofibers during electrospinning. The electrospun nanofibers were deposited on the metal plates of the two-parallel-metal-plate collector and suspended between the two parallel metal plates, forming a nanofiber mat on the void region of the collector^31^. As a representative example, the nanofiber mat on the void region was selected as the subject for the UTEN production system for the production of a uniform-thickness nanofiber mat. Furthermore, among the wide range of electrospinning materials, we chose PCL as for the UTEN process because of its excellent electrospinability and wide applicability, particularly in biomedical fields. Figure 2a,b show a two-parallel-metal-plate collector without and with the electrospun nanofiber mat, respectively. The nanofiber mat, which is indicated in white in Fig. 2b, was suspended between the two-parallel-metal-plate collectors. The production of the nanofiber was confirmed by investigating the magnified SEM image of the nanofiber mat (Fig. 2c).

### Real-time thickness measurement

**Figure 3:**
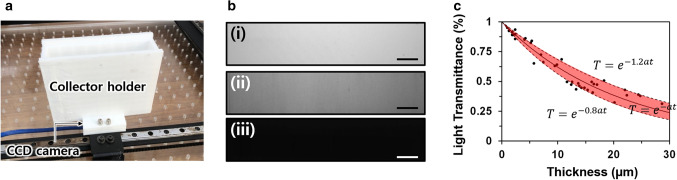
(**a**) Image of the 3D printed collector holder and CCD camera installed on the bottom of the collector holder. (**b**) Images captured by the CCD camera of the void region of the collector (i) without and (ii) with a nanofiber mat and (iii) with an opaque plate. (**c**) Curve of thickness vs. light intensity of the nanofiber mat. All scale bars indicate 1 cm.

The real-time thickness measurements were based on the conversion from the measured light intensity in the CCD camera images to the light transmittance of the nanofiber mat. The light emitted from the upper four bar LEDs passed through a nanofiber mat on the void region and reached the CCD camera, which was installed at the bottom of the collector. The light intensities at each pixel of the CCD camera image were evaluated, which corresponded to each position of the nanofiber mat at the collector, as the entire collector (100 × 21 mm) was projected onto the CCD camera with a pixel region of 900 × 170 pixels. To observe the entire collector region, the collector and CCD camera were set apart by 10 cm using the 3D-printed collector holder, as shown in Fig. 3a. The collector holder was composed of an opaque material and had a thick wall for blocking light from other sources. Figure 3b shows images of the CCD camera at the bottom of the collector without a nanofiber mat, with a nanofiber mat, and with an opaque plate. In the absence of the nanofiber mat, light passed through the void region between the two parallel metal plates without any obstacles. Thus, the CCD camera image without a nanofiber mat exhibited the highest light intensity due to the free light transmission (Fig. 3b(i)). In the case with a nanofiber mat, light was attenuated on passing through the nanofiber mat; thus, lower light intensities were measured by the CCD camera (Fig. 3b(ii)). Without the nanofiber mat on the void region of the collector, the light intensity reached a maximum, and as the thickness of the nanofiber mat increased, the light intensity decreased. To confirm the blockage of external light incident on the CCD camera, except for light passing through the nanofiber mat, we placed an opaque plate over the collector holder; an image with approximately zero light intensity was obtained by the CCD camera (Fig. 3b(iii)). The light transmittance of the nanofiber mat was calculated based on the measured light intensities with the nanofiber mat and reference light intensity without the nanofiber mat. As this procedure to measure the light transmittance of the nanofiber mat could be performed remotely without damaging the nanofiber mat, the light transmittance of the nanofiber mat could be measured in real time during electrospinning.

Figure 3c shows the relationship between light transmittance and the thickness of the nanofiber mat. The thickness of the nanofiber mat monotonically decreased with an increase in the light transmittance of the nanofiber mat. The attenuation of light passing through a homogenous medium is linearly proportional to the optical path length, which is described using the Beer–Lambert law^35^. Thus, light transmittance exponentially decreases with the optical path length or the thickness of the medium. For simplification, we assumed the nanofiber mat to be a homogenous medium, and data of the light transmittance and thickness of the nanofiber mat were fitted to the Beer–Lambert law using linear regression after taking the logarithm of the Beer–Lambert law. Thereafter, the attenuation coefficient, $a$, was calculated to be 0.04778 µm^−1^ using the fitted curve. The average relative error between the measured thickness and the estimated thickness calculated using the Beer–Lambert law with the obtained attenuation coefficient, $a$, was 18.84%. Moreover, approximately 60% of the measured thickness data were located between the fitting curves, with a ± 20% deviation of $a$ from 0.04778 µm^−1^, as highlighted in Fig. 3c. We believe that this error could be reduced using a higher bit-depth CCD camera and by adopting a more accurate method for measuring the thickness of the nanofiber mat. Finally, using the obtained attenuation coefficient, $a$, the UTEN production system estimated the thickness of the nanofiber mat in real time based on the measured light transmittance.

As the bit-depth of the CCD camera used in this study was 8-bit, including 256 levels, the minimum resolution of light transmittance was approximately 0.5%. Thus, the resolution of the real-time thickness measurement system was approximately 0.1 µm when the thickness of the nanofiber mat was 10 µm. However, when the thickness of the nanofiber mat was 100 µm, the resolution was approximately 10 µm because the light transmittance diminished exponentially with an increase in the thickness of the nanofiber mat. The UTEN production system adopted a two-parallel-metal-plate collector, which is used to fabricate nanofiber mats with thicknesses ranging from a few micrometers to several tens of micrometers. Thus, the proposed real-time thickness measurement system is used to appropriately evaluate the nanofiber mat fabricated by the UTEN production system. To apply this measurement technique to a thicker nanofiber mat (over several tens of micrometers) produced by electrospinning with a 2D flat collector or rotating drum, a higher bit-depth CCD camera (e.g., 12-bit depth or 16-bit depth) should be utilized in the real-time thickness measurement system, which could enhance the resolution of the thickness measurement.

### Deposition behavior of a nanofiber mat on a two-parallel-metal-plate collector

**Figure 4:**
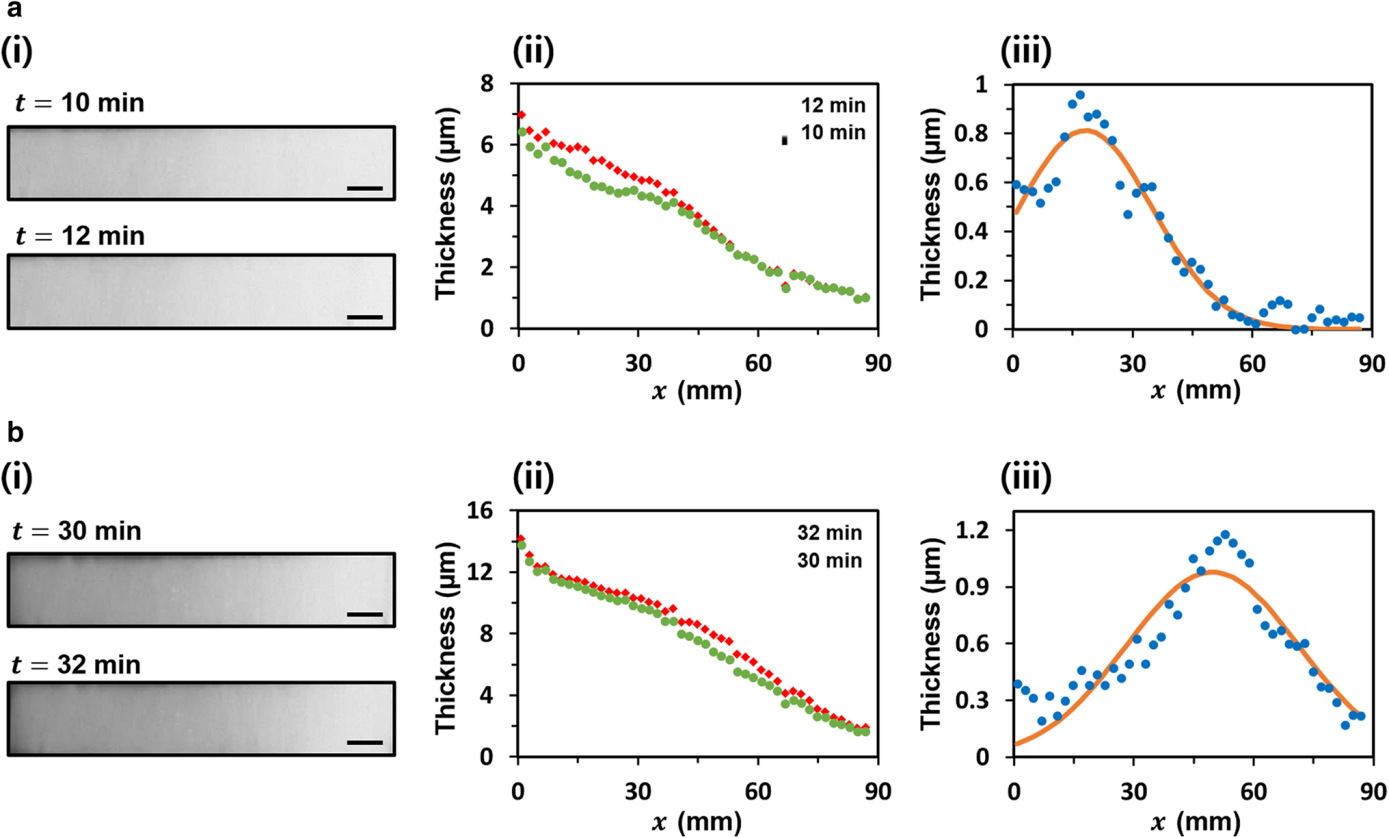
Deposition behavior of nanofiber mat in conventional electrospinning. (**a**) (i) bottom-view images and (ii) thickness along the horizontal axis of the nanofiber mats at 10 and 12 min. (iii) Amount of the deposited nanofiber mat from 10 to 12 min. (**b**) (i) bottom-view images and (ii) thickness along the horizontal axis of the nanofiber mats at 30 and 32 min. (iii) Amount of the deposited nanofiber mat from 30 to 32 min. All scale bars indicate 1 cm.

Figure 4a(i),b(i) show the images of the nanofiber mat from the CCD camera at 10, 12, 30, and 32 min. Along the horizontal axis, the variations in light transmittance of the nanofiber mat were noticeable, whereas along the vertical axis, the variations in light transmittance were insignificant. When nanofibers were suspended between the two parallel metal plates, the nanofibers tended to align along the vertical axis, which resulted in a lower non-uniformity of the nanofiber mat along the vertical axis. However, along the horizontal axis, the nanofiber mat was deposited randomly, and thus, a large variation was observed. Thus, we focused on reducing the non-uniformity of the nanofiber mat along the horizontal axis by simplifying it into a 1D problem.

Based on the Beer–Lambert law, the thicknesses of the nanofiber mat were calculated from the light transmittances along the center line of the horizontal axis at each time point, as shown in Fig. 4a(ii),b(ii). To model the deposition behavior of the nanofiber mat, the amount of the deposited nanofiber mat for 2 min at each time slot was calculated by subtracting the thickness from 12 to 10 min and that from 32 to 30 min, as shown in Fig. 4a(iii),b(iii). The deposition of the nanofiber mat was influenced by multiple factors, such as the electrostatic force related to the applied voltage and charge of deposited nanofibers, initial jet velocity governed by the flow rate, jetting regime and solvent evaporation governed by humidity and temperature, and particularly unpredictable bending instability due to high voltage. Thus, it is difficult to accurately predict the deposition of electrospun nanofibers during electrospinning. In this regard, the deposition behavior of the nanofiber mat at the 10- and 30-min time slots was empirically modeled using Gaussian distribution (Fig. 4a(iii),b(iii)). Based on the non-linear least-squares regression, the Gaussian distribution fitted to the amount of the deposited nanofiber mat for 2 min at each time slot showed a high value of the coefficient of determination (${\mathrm{r}}^{2}$ > 0.94), which statistically confirmed the validity of the modeling for a Gaussian distribution. On comparing the Gaussian distributions at 10 and 30 min, the center and standard deviation of the Gaussian distributions were found to be different, which implied that the deposition behavior of the nanofiber mat was continuously altered throughout electrospinning. Although we only discuss the different deposition behaviors at representative time slots of 10, 12, 30, and 32 min, such behavior was observed throughout electrospinning. These differences in deposition behavior would not be predictable in many cases owing to the complexity of the electrospinning process, particularly the instability near the Taylor cone. Owing to such unpredictability during the deposition of nanofibers, conventional electrospinning systems often produce a nanofiber mat with non-uniform thickness.

### Thickness feedback control

**Figure 5:**
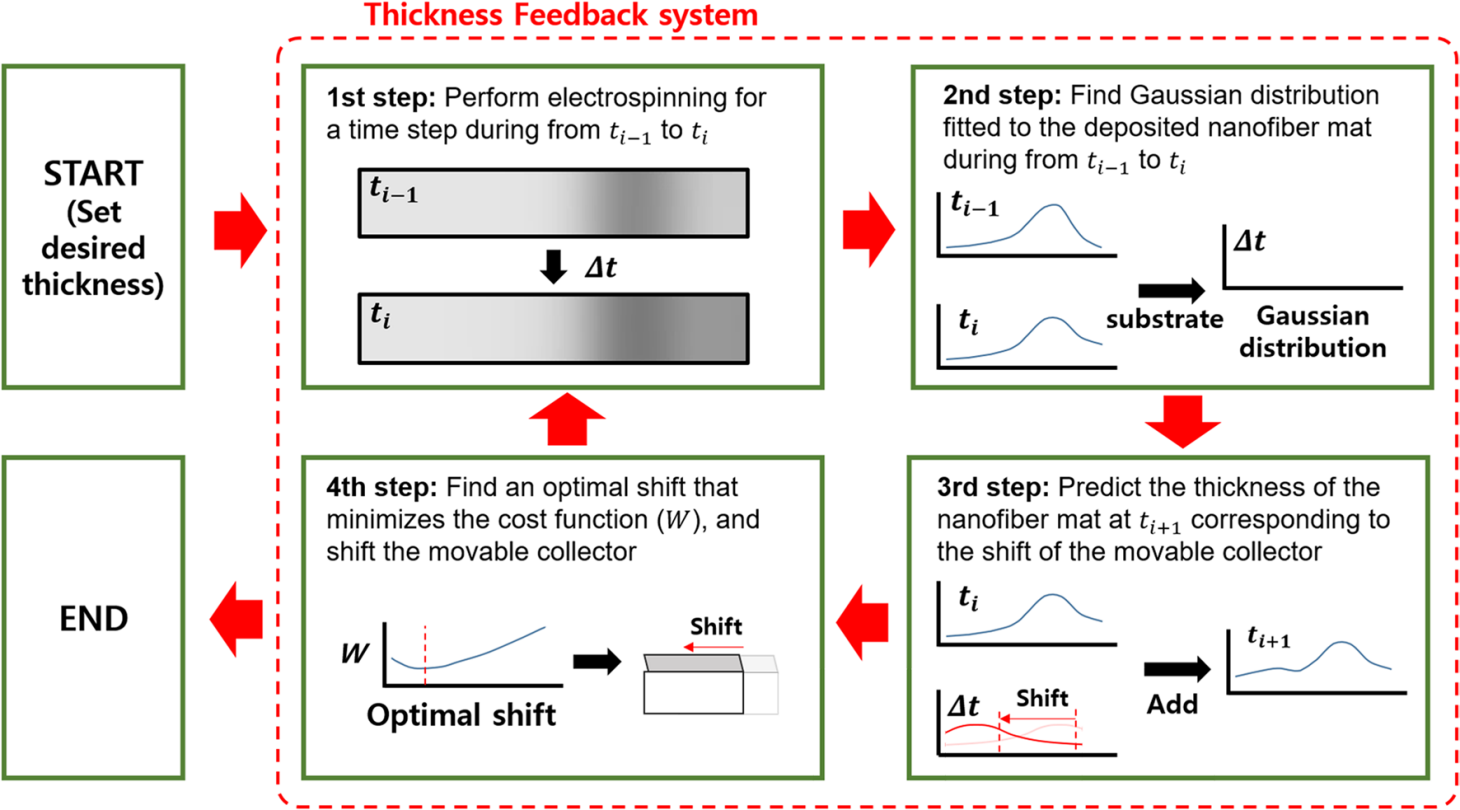
Flowchart of the thickness feedback control algorithm.

Figure 5 shows a detailed flowchart of the thickness feedback control algorithm used to produce a uniform-thickness nanofiber mat. The first step of the thickness feedback control algorithm involved performing the electrospinning process for a given time step ($\Delta t$) in order to deposit a nanofiber mat on the movable collector. The notable feature of this thickness feedback control algorithm is the large time step of more than 1 min. To model the deposition behavior of a nanofiber mat during this time step, a minimum amount of nanofibers needs to be deposited on the movable collector. Empirically, electrospinning for at least 1 min was required to deposit an adequate amount of nanofibers for modeling the deposition behavior. Thus, in this study, we set the time step to 2 min. The second step involved calculating the amount of nanofiber mat deposited from the previous time (${t}_{i-1}$) to the current time (${t}_{i}$) by subtracting the thickness of the nanofiber mat at ${t}_{i}$ from that at ${t}_{i-1}$. Subsequently, the thickness of the nanofiber mat deposited from ${t}_{i-1}$ to ${t}_{i}$ was fitted with a Gaussian distribution using non-linear least-square regression. The third step involved predicting the thickness of the nanofiber mat for the next time (${t}_{i+1}$) corresponding to the shift of the movable collector. To predict the thickness of the nanofiber mat for ${t}_{i+1}$, we assumed that (1) the nanofiber mat deposited from ${t}_{i}$ to ${t}_{i+1}$ had the same Gaussian distribution from ${t}_{i-1}$ to ${t}_{i}$, based on the empirical observation that the deposition behavior remains unchanged within a short period of time, and (2) the Gaussian distribution shifts with the same distance and in the opposite direction as the movable collator, a simplification of the deposition behavior of the nanofiber mat. Thus, the thickness of the nanofiber mat at ${t}_{i+1}$ was predicted by adding the thickness of the nanofiber mat at ${t}_{i}$ and the predicted Gaussian distribution corresponding to the shift of the movable collector. The fourth step involves finding an optimal shift of the movable collector to minimize the variance and error of the thickness of the nanofiber mat at ${t}_{i+1}$ corresponding to the shift of the movable collector. Hence, we defined the cost function for the addition of a normalized mean squared error and variance of the thickness of the nanofiber mat for ${t}_{i+1}$ corresponding to the shift of the movable collector. The cost function $W(s)$ is defined as follows:

1 $$W\left(s\right)=\frac{S{E}_{i+1}\left(s\right)}{S{E}_{i}}+\frac{{\sigma }_{i+1}^{2}\left(s\right)}{{\sigma }_{i}^{2}}$$

where $s$ is the shift of the movable collector, ${SE}_{i+1}(s)$ is the sum of the squared error between the desired thickness and predicted thickness corresponding to $s$, ${SE}_{i}$ is the sum of the squared error between the desired thickness and current thickness, ${\sigma }_{i+1}^{2}(s)$ is the variance of the predicted thickness corresponding to $s$, and ${\sigma }_{i}^{2}$ is the variance of the current thickness. The first term is the sum of the normalized mean squared error between the target thickness and predicted thickness for all positions. Minimization of the first term reduced the error between the target thickness and actual thickness. The second term is the normalized variance of the predicted thickness. Minimization of the second term would improve the uniformity of the nanofiber mat. Thus, moving the collector to an optimal position that minimizes the cost function of the first and second terms would simultaneously achieve a reduction in the error and an increase in the uniformity. After determining an optimal position and moving the collector to the optimal position, the entire process was repeated, from the first step of electrospinning, until the optimal shift increased the cost function.

### Numerical and experimental validation of the UTEN production system

**Figure 6:**
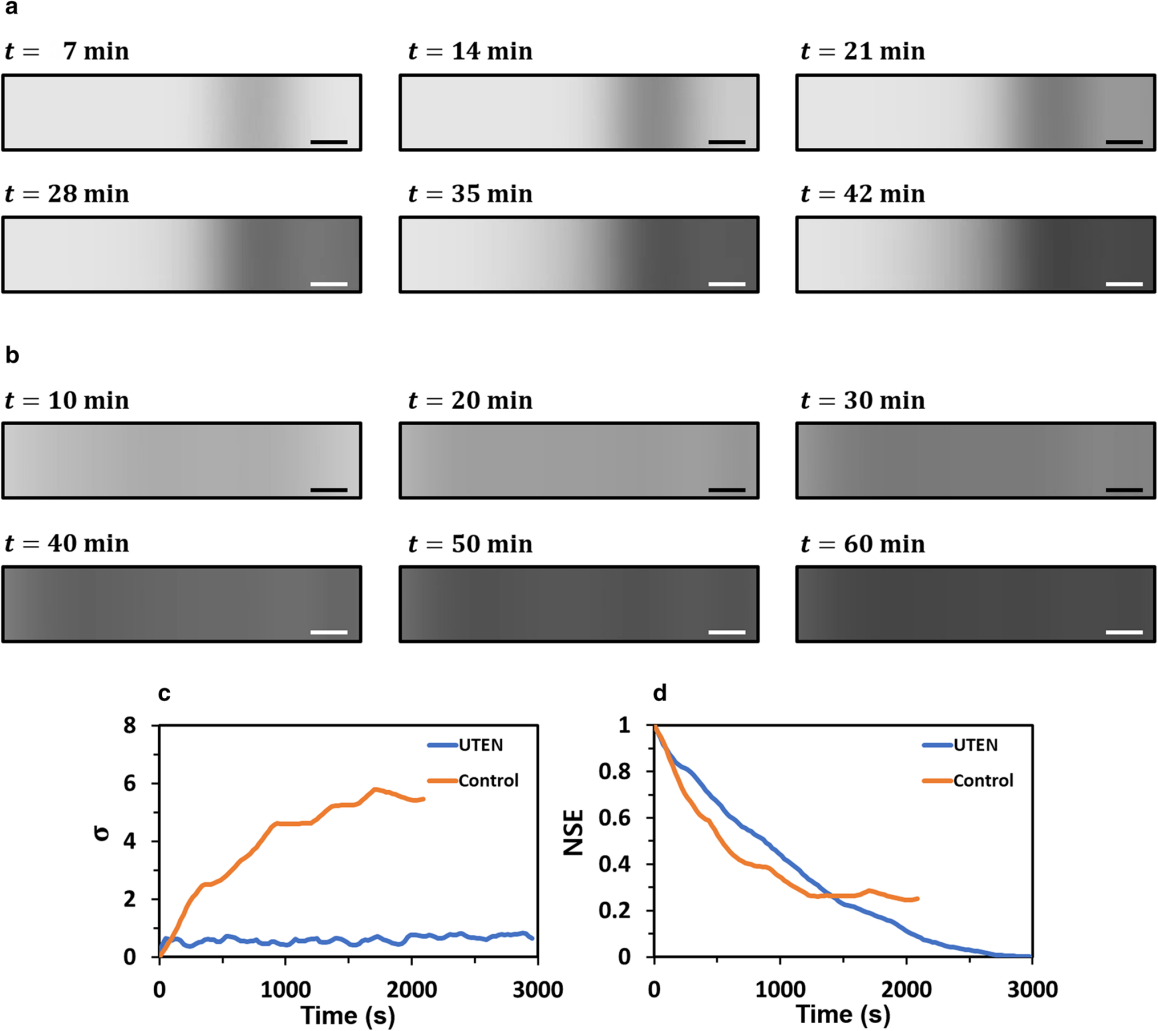
Simulation results. The bottom-view images of the nanofiber mat simulated based on (**a**) conventional electrospinning system and (**b**) UTEN production system over time. (**c**) Comparison of standard deviation $(\upsigma )$ and (**d**) normalized squared error (NSE) between the conventional electrospinning and UTEN production system. All scale bars indicate 1 cm.

Figure 6a shows the simulation results of the bottom-view images of a nanofiber mat produced by electrospinning over time without applying the thickness feedback control algorithm of the UTEN production system. As shown in Fig. 6a, from 7 to 14 min, the right portions of the bottom-view images became darker following a Gaussian distribution. After 21 min, the Gaussian distribution shifted because of the simulated randomness of the nanofiber deposition of conventional electrospinning. Figure 6b shows the bottom-view images of a nanofiber mat simulated based on the UTEN production system with the thickness feedback control algorithm. The bottom-view images becomes darker with time because of the deposition of the nanofiber mat. Unlike that in Fig. 6a, this nanofiber mat darkens uniformly, implying that the nanofiber mat produced via the UTEN production system had a uniform thickness.

Figure 6c shows the standard deviation of the thickness of the nanofiber mat over time with and without the thickness feedback control algorithm. The standard deviation indicates the degree of non-uniformity of the nanofiber mat along the horizontal axis. The standard deviation of the nanofiber mat in the UTEN production system was less than that of the nanofiber mat obtained via the conventional electrospinning system (Fig. 6c). The standard deviation of the nanofiber mat from the UTEN production system was 8 times less than that of the conventional electrospinning system. Figure 6d shows that the normalized mean squared errors in the two cases decreased at a similar rate. However, the nanofiber mat produced without the thickness feedback control algorithm exhibits a higher degree of non-uniformity that that produced with the thickness feedback control algorithm. In conclusion, the simulation results showed that the UTEN production system algorithm is effective in reducing the non-uniformity of the nanofiber mat.

**Figure 7:**
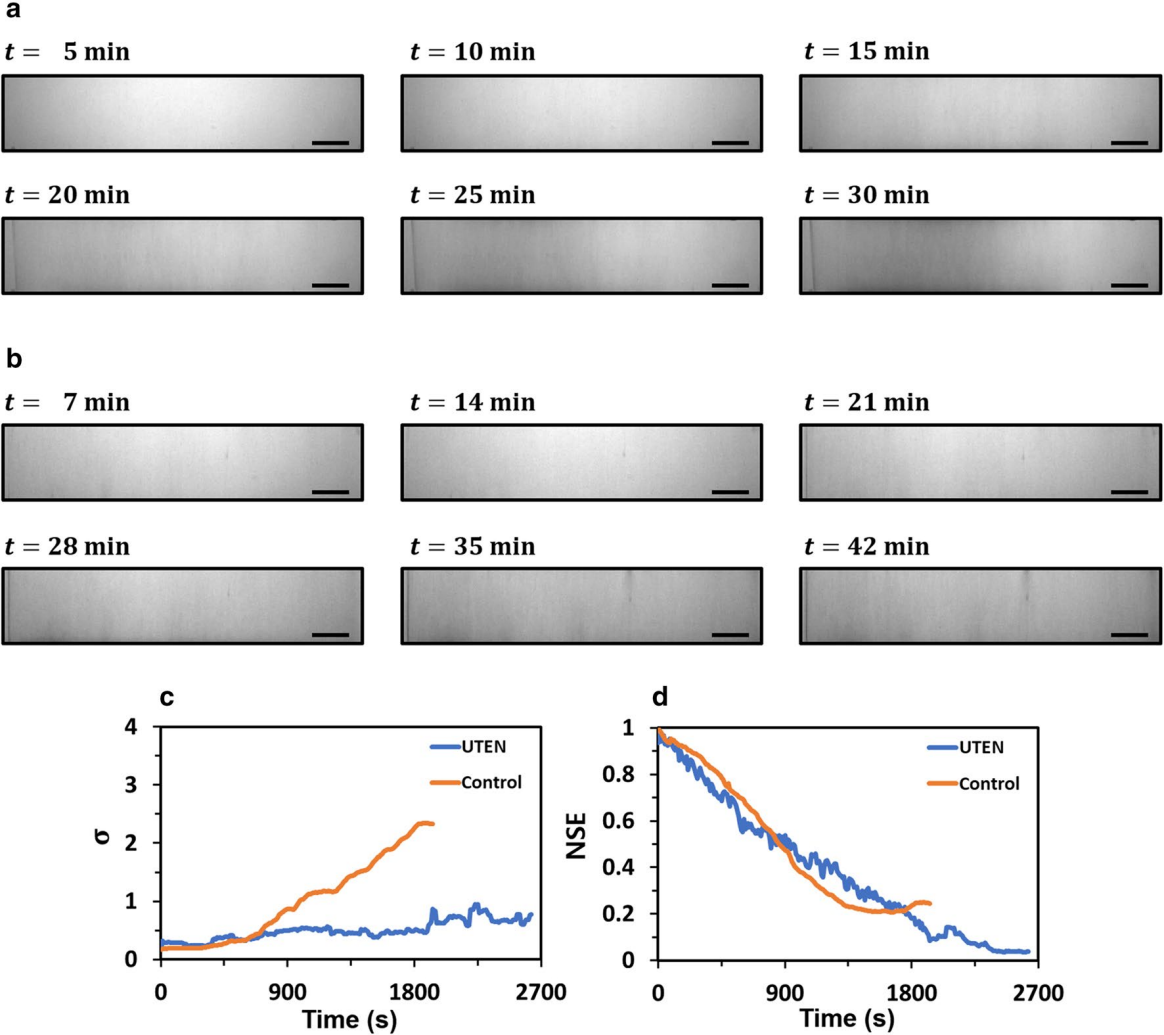
Experimental results. The bottom-view images of the nanofiber mat fabricated using (**a**) conventional electrospinning system and (**b**) UTEN production system over time. (**c**) Comparison of standard deviation $(\upsigma )$ and (**d**) normalized squared error (NSE) between the conventional electrospinning and UTEN production system. All scale bars indicate 1 cm.

The performance of the UTEN production system, which was verified by simulation, was also experimentally validated. Figure 7a shows the bottom-view images of a nanofiber mat fabricated using the conventional electrospinning system with respect to time. The brightness of the images exhibited non-uniformity, and the left portion of the image exhibited a gradient following a Gaussian distribution, as previously identified by the numerical simulation. Figure 7b shows the bottom-view images of a nanofiber mat that was experimentally fabricated using the UTEN production system with the thickness control algorithm. The images in Fig. 7b became darker over time and maintained uniformity. This result shows that the UTEN production system with the thickness feedback control algorithm enabled the production of a nanofiber mat with a uniform thickness, similar to the numerical simulation results. Figure 7c,d show the standard deviation and normalized squared errors of the thickness of the nanofiber mat fabricated using the conventional electrospinning system and that fabricated using the UTEN production system, respectively. While the normalized squared errors of the two systems decreased at a similar rate, as the nanofibers accumulated at a constant rate, the final normalized squared error of the nanofiber mat from the UTEN production system was significantly lower than that obtained via the conventional electrospinning system. Furthermore, the standard deviation of the nanofiber mat produced using the conventional electrospinning system and that of the nanofiber mat fabricated using the UTEN production system differed significantly, by a factor of more than 3. The standard deviation of the thickness of the nanofiber mat fabricated using the UTEN production system remained low during electrospinning, whereas the standard deviation of the nanofiber mat produced by the conventional system increased. This indicates that the thickness of the nanofiber mat from the UTEN production system was more uniform than that obtained via the conventional electrospinning system and that the UTEN production system was considerably effective in generating a nanofiber mat with uniform thickness.

## Conclusion

In this study, a UTEN production system was developed to produce a uniform-thickness nanofiber mat. The UTEN production system was based on the Beer–Lambert Law, which states that light transmittance has an exponential relationship with the thickness of the nanofiber mat; this was used to determine the current thickness of the nanofiber mat. To minimize the variance and normalized squared error of the thickness of the nanofiber mat, the movable collector was shifted to an optimal position that enabled the uniform deposition of the nanofibers. The performance of the UTEN production system was verified through numerical simulation and experiments. As a result, it was confirmed that the uniformity in the thickness of the nanofiber mat was drastically improved. Nevertheless, the current study involves a limitation, that is, the proposed UTEN production system is only applied to the 1D case of two-parallel-metal-plate collectors. If the real-time thickness measurement and thickness feedback control algorithm is made applicable for the case of a 2D flat collector, the UTEN system can then be employed in 2D collector systems, enabling a broad range of applications.

## Supplementary information

Supplementary Information.
