# Supplementary material for: Uniform-thickness electrospun nanofiber mat production system based on real-time thickness measurement
Source: Sci Rep. 2020 Nov 30;10:20847. doi: 10.1038/s41598-020-77985-0 (PMC7705742; doi:10.1038/s41598-020-77985-0)
Supplement: Supplementary file 1 — Supplementary Information. [file 41598_2020_77985_MOESM1_ESM.docx]

Supplementary Information for

**Uniform-thickness electrospun nanofiber mat production system based on real-time thickness measurement**

Hyun Il Ryu^1,+^, Min Seok Koo^1,+^, Seok Jun Kim^1^, Song Kil Kim^1^, Young-Ah Park^2,^*, Sang Min Park^1,^**

^1^School of Mechanical Engineering, Pusan National University, Busan, 46241, South Korea

^2^Division of Cardiology, Inje University, Busan Paik Hospital, Busan, 47392, South Korea

*corresponding author: [sangmin.park@pusan.ac.kr](mailto:smkds@postech.ac.kr)

**corresponding author: youngcv84@naver.com

^+^These authors are equally contributed to this work.

**1. Bar LEDs and a light diffuser panel**


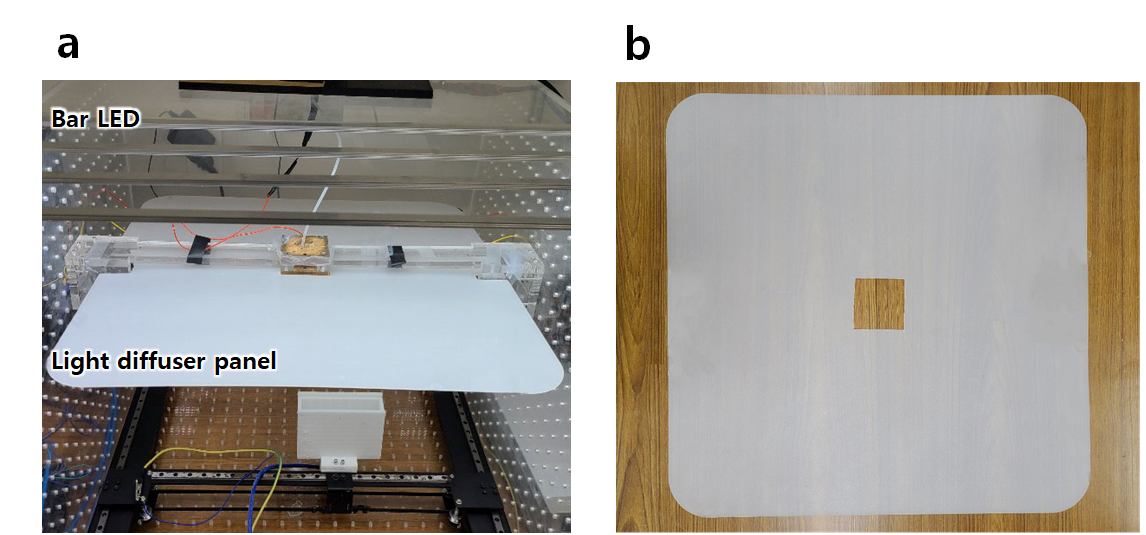


**Figure S1.** (a) Configuration of 4 bar LEDs and a light diffuser panel (b) A photo of a light diffuser panel

**2. Cross-sectional image of the nanofiber mat embedded in PDMS**


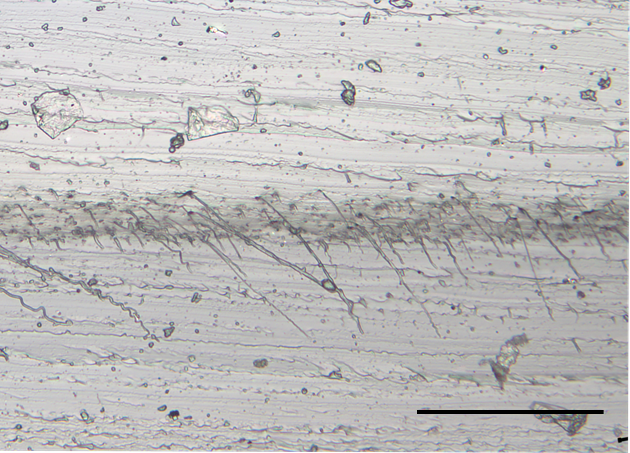


**Figure S2.** Cross-sectional image of the nanofiber mat embedded in PDMS. The scale bar indicates 100 µm.

**1. Bar LEDs and a light diffuser panel**


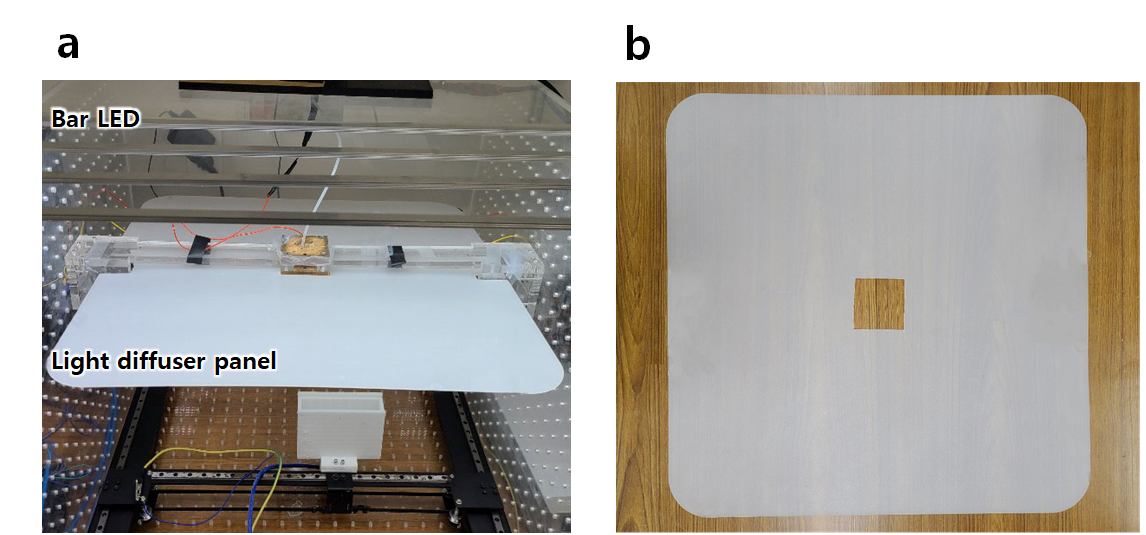


**Figure S1.** (a) Configuration of 4 bar LEDs and a light diffuser panel (b) A photo of a light diffuser panel
